# Prevalence and socioeconomic correlates of overweight and obesity among Pakistani primary school children

**DOI:** 10.1186/1471-2458-11-724

**Published:** 2011-09-25

**Authors:** Muhammad Umair Mushtaq, Sibgha Gull, Hussain Muhammad Abdullah, Ubeera Shahid, Mushtaq Ahmad Shad, Javed Akram

**Affiliations:** 1 Ubeera Memorial Research Society, Allama Iqbal Medical College, Lahore, 54000 Punjab, Pakistan; 2 District Health Office Nankana Sahib, Punjab Department of Health, Nankana Sahib, 39100 Punjab, Pakistan; 3 King Edward Medical University, Lahore, 54000 Punjab, Pakistan

## Abstract

**Background:**

Childhood obesity is becoming an equally challenging, yet under-recognized, problem in developing countries including Pakistan. Children and adolescents are worst affected with an estimated 10% of the world's school-going children being overweight and one quarter of these being obese. The study aimed to assess prevalence and socioeconomic correlates of overweight and obesity, and trend in prevalence statistics, among Pakistani primary school children.

**Methods:**

A population-based cross-sectional study was conducted with a representative multistage cluster sample of 1860 children aged 5-12 years in Lahore, Pakistan. Overweight (> + 1SD) and obesity (> + 2SD) were defined using the World Health Organization child growth reference 2007. Chi-square test was used as the test of trend. Linear regression was used to examine the predictive power of independent variables in relation to BMI. Logistic regression was used to quantify the independent predictors for overweight and adjusted odds ratios (aOR) with 95% confidence intervals (CI) were obtained. All regression analyses were controlled for age and gender and statistical significance was considered at P < 0.05.

**Results:**

Seventeen percent (95% CI 15.4-18.8) children were overweight and 7.5% (95% CI 6.5-8.7) were obese. Higher prevalence of obesity was observed among boys than girls (P = 0.028), however, there was no gender disparity in overweight prevalence. Prevalence of overweight showed a significantly increasing trend with grade (P < 0.001). Children living in the urban area with high socioeconomic status (SES) were significantly at risk for being overweight and obese (both P < 0.001) as compared to children living in the urban area with lower SES and rural children. Being in higher grade (aOR 2.39, 95% CI 1.17-4.90) and living in the urban area with higher SES (aOR 18.10, 95% CI 10.24-32.00) independently predicted the risk of being overweight.

**Conclusion:**

Alarmingly rapid rise in overweight and obesity among Pakistani primary school children was observed, especially among the affluent urban population. The findings support the urgent need for National preventive strategy for childhood obesity and targeted interventions tailored to local circumstances with meaningful involvement of communities.

## Background

Overweight and obesity are a global epidemic, with 1 billion overweight people, of whom 300 million are obese, and at least 2.6 million die each year as a result of being overweight or obese [1]. The 2004 World Health Assembly at Geneva called for specific action to halt the epidemic that is now penetrating the poorest nations in the world, especially amongst the urban [2,3]. Children and adolescents are worst affected with an estimated 10% of the world's school-going children being overweight and one quarter of these being obese [4,5]. Childhood obesity adversely affects physiological and psychosocial well-being; significantly increases the likelihood for adult obesity; results in non-communicable diseases (NCDs) like diabetes, cardiovascular disease and cancer; and leads to increased mortality and morbidity, heavy health expenditures and reduced social status [5-11].

Childhood obesity is becoming an equally challenging, yet under-recognized, problem in the developing countries including Pakistan [10-12]. Targeted interventions, tailored to local circumstances and involving communities, should begin early in life [13,14]. Globally, prevalence of childhood overweight and obesity among school-going children has been extensively explored [15-21], and many interventions have been implemented for the prevention of childhood obesity in early school years [22]. In Pakistan, however, it has been neglected and only two surveys with statistically representative sample have been conducted including the National Health Survey of Pakistan (NHSP) 1990-94 and the Karachi survey 2004-05 that reported prevalence of overweight as 3% and 5.7% respectively among urban school-aged children [23]. Routine surveillance on prevalence of childhood overweight and obesity has not been conducted in Pakistan, and magnitude of the problem can be determined only through special studies. In 2009-10, a cross-sectional survey titled the Nutritional Assessment among School-going Children in Lahore, Pakistan (NASCL) was conducted among primary school children aged 5-12 years. In Pakistan, it was the third representative sample of nutritional assessment among school-aged children and first large-scale study to explore factors associated with nutritional status. Prevalence and socioeconomic correlates of overweight and obesity, and trend in prevalence statistics, among Pakistani primary school children is the subject of current paper.

## Methods

### Design, setting and sample

This was a population-based cross-sectional study among primary school children aged 5-12 years in Lahore, Pakistan. Lahore is the capital of Pakistan's most populous province Punjab and a metropolis with multiethnic populations. It has a population of about 9 million, including about 2.5 million primary school children aged 5-12 years, and 81% of the population resides in the urban area (Administrative data, Government of the Punjab, 2010).

A stratified multistage cluster sample of 1860 children aged 5-12 years in twelve primary schools of City District Lahore was enrolled. The same sampling design has been used previously in nutritional assessment surveys [15-19]. Stratified sampling, based on the population and educational system characteristics, was used to have proportionate representation of gender, area of residence and socioeconomic status (SES). The list of all the public and private primary schools in Lahore was provided by the Punjab Department of Education. The listed schools were stratified according to the geographic area and monthly fee structure of the schools into following four strata: a) urban with high SES (urban area and fee > 2500 PKR), b) urban with middle SES (urban area and fee = 1000-2500 PKR), c) urban with low SES (urban area and fee < 1000 PKR), and d) rural with low/disadvantaged SES (rural area and fee ~100 PKR or free). The former two strata included private (including public-private mix) schools and the later two strata included public schools. In Pakistan, public schools cater low SES urban and rural children while high and middle SES urban children are educated in private and public-private mix schools. Three schools were selected at random from each stratum and contacted by the Departments of Education and Health to participate voluntarily in the study. If the school administration refused to participate, the next school was selected randomly from the respective stratum. For each school, a list of all classes in the five grades (one to five) was obtained and one class in each grade was randomly selected. In this way, 60 classes, five from each school, were selected. For each of the selected classes, first 31 children on the class attendance register, present on data collection day and aged 5-12 years, were included in the study. Children suffering from any known metabolic syndrome (like Prader-Willi syndrome) and children not willing or unable to participate in the study were excluded.

Sample size was calculated using Epi Info 6.04d (US Centers for Disease Control and Prevention, 2004) with a confidence (1-α) of 95%, anticipated prevalence of 5%, and margin of error of ±1. The minimum sample size calculated was 1823 and a sample of 1860 was deemed sufficient.

### Data Collection

The most frequently used measure for obesity is body mass index (BMI), defined as weight (kg)/height squared (m^2^), and BMI-for-age is the anthropometric index of relative weight recommended by the international expert committees [24]. For data collection, sampled schools were visited on pre-arranged dates in summer 2009 by teams of trained senior medical students lead by the Principal Investigator. Health education of children and teachers was also carried out after data collection in the respective school.

Analogue physician health scales were used to measure height and weight [25]. All instruments were standardized before the examination and the balances were zero calibrated. Height and weight were measured without shoes and in light summer school uniform. Timing of the measurements was in the mornings or early afternoons. Height measurement was in centimeters (cm) and weight was measured in kilogram (kg) with a range of 0-160 kg. Height and weight were measured to the nearest 0.1 cm and 0.5 kg respectively. Feet were placed together with heels, buttocks and shoulder blades against the stick and head in the Frankfurt plane with anthropometric square.

For each of the selected classes in the sample, demographic information of all officially enrolled students was obtained before data collection, including gender, date of birth and residential address. Demographic information of students not found on official rosters but currently enrolled in that class was obtained from the classroom teachers. Quality control measures and good practices included training of the surveyors, pre-testing the processes and materials and field monitoring of the data collection. Timely availability of data collection instruments, meeting of survey teams at the end of everyday to share experiences and submission of completed forms and troubleshooting field problems was ensured.

The informed consent statement was printed on the form. Verbal informed consent for the child to participate in the study was taken from class teachers and school heads considering them as guardians. As the study involves no invasive procedure, verbal informed consent was deemed sufficient. The study was approved by the Ethical Review Board of Allama Iqbal Medical College, Lahore. Permissions to conduct the study were granted by the Punjab Departments of Education and Health, and the sampled schools.

### Statistical Analysis

Data was entered and analyzed by manual and computerized checking using SPSS version 18.0 (SPSS Inc. Chicago IL, United States, 2009). Age was calculated to the precise day by subtracting the date of birth from the date of examination. Anthropometric measures including weight, height and BMI were presented with the means and standard deviation (SD). The z-score values for BMI-for-age were calculated by using the World Health Organization's software, AnthroPlus, for assessing growth of the world's children and adolescents (WHO, 2009). Overweight (> + 1SD) and obesity (> + 2SD) were defined using the WHO child growth reference 2007 [26,27]. Prevalence of overweight and obesity was also presented according to the International Obesity Task Force (IOTF) cut-offs [28]. Grade was used in the analyses instead of age for more uniform representation, consistent with previous studies [15,16].

Crude odds ratios (OR) with 95% confidence intervals (CI) were calculated by univariate analysis to compare prevalence of overweight and obesity among study variables. Bivariate analysis, using chi-square test as the test of trend, was conducted to compare differences in prevalence of overweight and obesity among study variables. Linear regression was used to examine the predictive power of the independent variables (grade and area/SES) in relation to BMI (dependent variable). Logistic regression was used to estimate the simultaneous effect of several determinants on a dichotomous (yes/no) outcome. Grade and area/SES were entered into the multivariate model concurrently to quantify their independent importance for risk of being overweight and adjusted odds ratios (aOR) were obtained. All regression analyses were controlled for age and gender. Statistical significance was considered at P < 0.05 and all tests were 2-sided.

## Results

The study included a sample of 1860 primary school children aged 5-12 years. The male-female ratio was 1.11 with 52.5% boys and 47.5% girls. The sample involved 20% children from each grade and 25% children from each area and SES stratum. Seventy-five percent children were urban and 25% children were rural. The median age (range) was eight (5-12) years and the mean age (SD) was 8.49 (1.81) years.

The means (SD) for height, weight and BMI were 128.4 (11.4) cm, 26.9 (8.5) kg and 20.7 (5.02) kg/m^2 ^respectively [Table 1]. Seventeen percent (95% CI 15.4-18.8) children were overweight and 7.5% (95% CI 6.5-8.7) were obese. Severe obesity was observed in 2% (95% CI, 1.3-2.6) children. According to the IOTF cut-offs, overweight and obesity prevalence was 33% (95% CI 31.1-35.3) and 24% (95% CI 22.4-26.2) respectively [Table 2].

**Table 1:** Mean and standard deviation (SD) for height, weight and BMI of primary school children in Lahore, Pakistan (n = 1860)

Characteristics	n	Height (cm)	Weight (kg)	**BMI (kg/m**^**2**^**)**
**Boys (n = 977)**				
5 years (61-71 months)	84	113.7 (7.3)	19.9 (4.6)	17.4 (3.0)
6 years (72-83 months)	161	118.3 (5.9)	21.6 (5.0)	18.2 (3.7)
7 years (84-95 months)	160	122.9 (8.0)	23.5 (5.1)	19.0 (3.4)
8 years (96-107 months)	158	128.7 (7.6)	26.9 (5.9)	20.8 (3.8)
9 years (108-119 months)	161	134.2 (8.1)	29.7 (7.6)	21.9 (4.7)
10 years (120-131 months)	147	138.4 (8.0)	33.3 (9.5)	23.9 (5.8)
11 years (132-143 months)	69	138.6 (7.7)	31.8 (6.8)	22.8 (4.2)
12 years (144-155 months)	37	140.0 (8.3)	31.8 (7.3)	22.6 (4.1)
**Girls (n = 883)**				
5 years (61-71 months)	72	115.4 (7.3)	19.3 (3.2)	16.6 (2.0)
6 years (72-83 months)	143	119.1 (7.6)	21.0 (4.9)	17.6 (3.3)
7 years (84-95 months)	157	124.0 (6.3)	24.0 (5.5)	19.3 (3.7)
8 years (96-107 months)	159	128.1 (7.1)	26.4 (6.8)	20.5 (4.4)
9 years (108-119 months)	151	133.3 (7.8)	30.4 (8.2)	22.7 (5.3)
10 years (120-131 months)	120	138.4 (9.3)	33.3 (10.1)	23.9 (6.1)
11 years (132-143 months)	62	143.3 (9.6)	36.5 (11.0)	25.2 (6.4)
12 years (144-155 months)	19	146.0 (9.4)	36.4 (9.9)	24.8 (5.7)

**Table 2:** Prevalence of overweight and obesity among primary school children in Lahore, Pakistan (n = 1860)

			WHO 2007		IOTF
			
Characteristics	n	Mean BMI (SD)	% (95% CI)	Mean BMI-for-age z-score (SD)	% (95% CI)
Severely obese	36	33.7 (7.1)	1.9 (1.3-2.6) ^a^	3.7 (0.7)	
Obese	140	31.3 (5.7)	7.5 (6.3-8.7) ^b^	2.8 (0.7)	24.3 (22.4-26.2) ^d^
Overweight	316	28.5 (5.3)	17.0 (15.4-18.8) ^c^	2.0 (0.8)	33.2 (31.1-35.3) ^e^
Total sample	1860	20.7 (5.0)		-0.3 (1.5)	

^a^> + 3SD, ^b^> + 2SD, ^c^> + 1SD of World Health Organization (WHO) 2007 BMI-for-age reference

^d, e^International Obesity Task Force (IOTF) cut-offs for obesity and overweight respectively

**Figure 1:**
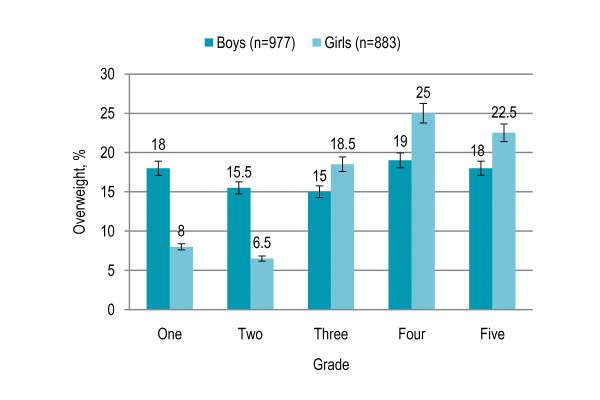
Grade- and gender- specific prevalence (with confidence interval bars) of overweight among primary school children in Lahore, Pakistan (n = 1860)

**Figure 2:**
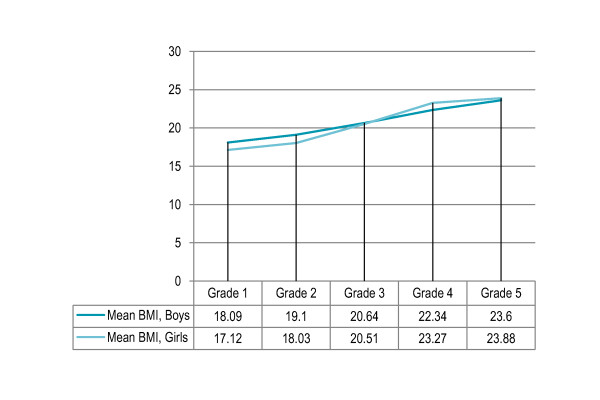
Grade- and gender- specific mean BMI among primary school children in Lahore, Pakistan (n = 1860)

More boys were overweight (17%) than girls (16.5%) but the association was not statistically significant, however, obesity prevalence was significantly higher among boys as compared to girls (9% versus 6%, P = 0.028). Prevalence of overweight showed a significantly increasing trend with grade (P < 0.001), with 14.5% and 11% overweight children in grades one and two respectively that increased to 17% overweight children in grade three and to 22% and 20% overweight children in grades four and five respectively. Similar trend was noted in the respective age groups. Grade- and gender-specific prevalence of overweight showed a significant positive association with grade among girls (P < 0.001) but the relation was not significant among boys. More boys were overweight in grades one and two while more girls were overweight in grades three to five [Figure 1]. The mean BMI was higher in boys in grades one and two as compared to girls while in grades four and five a higher BMI in girls was observed than boys [Figure 2].

**Figure 3:**
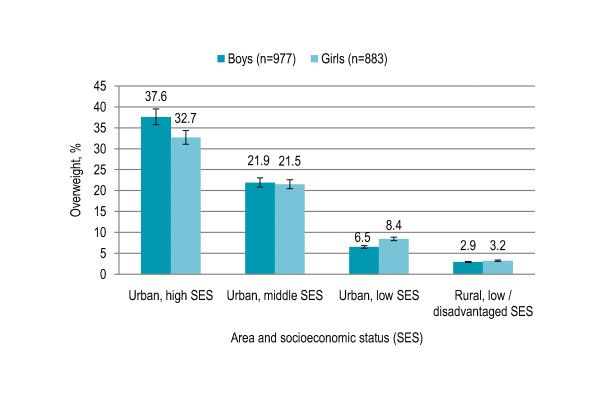
**Gender-specific prevalence (with confidence interval bars) of overweight by area and socioeconomic status among primary school children in Lahore, Pakistan (n = 1860)**.

Children living in the urban area with high SES were significantly at risk for being overweight and obese (both P < 0.001) than children living in the urban area with lower SES and rural children. Prevalence of overweight and obesity was 35.5% and 18% respectively in the urban children with high SES that decreased to 22% and 10% respectively in the urban children with middle SES and to 8% and 2% respectively in the urban children with low SES. Only 3% children living in the rural area (low/disadvantaged SES) were overweight and only 0.6% children were obese. Overweight and obesity prevalence was 5% and 1% respectively in public schools that increased to 29% and 14% respectively in private (including private-public mix) schools (P < 0.001) [Table 3]. No gender disparity was observed in prevalence of overweight in the rural area and the urban area with middle SES, however, more boys were overweight in the urban area with high SES and more girls were overweight in the urban area with low SES [Figure 3].

**Table 3:** Sociodemographic factors associated with overweight and obesity in Lahore, Pakistan (n = 1860)

	Total Sample	Over weight (n = 316)		Obese (n = 140)	
	
Characteristics	n (%)	n (%)	P value	n (%)	P value
**Gender**			0.620		0.028
Boys	977 (52.5)	170 (17.4)		86 (8.8)	
Girls	883 (47.5)	146 (16.5)		54 (6.1)	
**Grade**			< 0.001		0.030
One	372 (20.0)	54 (14.5)		35 (9.4)	
Two	372 (20.0)	41 (11.0)		18 (4.8)	
Three	372 (20.0)	63 (16.9)		26 (7.0)	
Four	372 (20.0)	82 (22.0)		38 (10.2)	
Five	372 (20.0)	76 (20.4)		23 (6.2)	
**Age**			0.003		0.093
5-6 years (60-83 months)	460 (24.7)	53 (11.5)		36 (7.8)	
7-8 years (84-107 months)	634 (34.1)	117 (18.5)		44 (6.9)	
9-10 years (108-131 months)	579 (31.1)	114 (19.7)		53 (9.2)	
11-12 years (132-155 months)	187 (10.1)	32 (17.1)		07 (3.7)	
**Area and socioeconomic status (SES)**			< 0.001		< 0.001
Urban, high SES	465 (25.0)	165 (35.5)		82 (17.6)	
Urban, middle SES	465 (25.0)	101 (21.7)		47 (10.1)	
Urban, low SES	465 (25.0)	36 (7.7)		08 (1.7)	
Rural, low/disadvantaged SES	465 (25.0)	14 (3.0)		03 (0.6)	
**School type**			< 0.001		< 0.001
Public	930 (50.0)	50 (5.4)		11 (1.2)	
Private (and public-private mix)	930 (50.0)	266 (28.6)		129 (13.9)	

In linear regression analysis controlled for age and gender, grade showed a significant positive association with BMI (regression coefficient 0.65, 95% CI 0.41 to 0.89) while area/SES showed a significant inverse association with BMI (regression coefficient -1.54, 95% CI -1.71 to -1.38) [Table 4]. Multiple logistic regression analysis was adjusted simultaneously for age, gender, grade and area/SES. Children in grade four were significantly more likely to be overweight than children in grade one (aOR 2.39, 95% CI 1.17-4.90) and children living in the urban area with high SES were significantly more likely to be overweight than those living in the rural area with low/disadvantaged SES (aOR 18.10, 95% CI 10.24-32.00) [Table 5].

**Table 4:** Linear regression analysis of factors associated with BMI among primary school children in Lahore, Pakistan (n = 1860) ^a,b^

Characteristics	Regression coefficient (95% CI)	Standard error	P value
(Constant)	15.04 (13.73 to 16.35)	0.67	< 0.001
Grade ^c^	0.65 (0.41 to 0.89)	0.12	< 0.001
Area and SES ^d^	-1.54 (-1.71 to -1.38)	0.08	< 0.001

^a^The model is adjusted for age and gender

^b^R^2 ^= 0.355

^c^Grades: 1-5 grades

^d^Area and socioeconomic status (SES) strata: 1. Urban with high SES, 2. Urban with middle SES, 3. Urban with low SES, and 4. Rural (low/disadvantaged SES)

**Table 5:** Logistic regression model of factors associated with overweight (including obesity) among primary school children in Lahore, Pakistan (n = 1860) ^a^

	Over-weight, including obese (n = 316)			
	
Characteristics	Crude OR (95% CI)	P Value	**Adjusted OR (95% CI) **^**a**^	P Value
**Grade**				
One	Reference	-	Reference	-
Two	0.75 (0.48-1.15)	0.187	0.80 (0.49-1.32)	0.379
Three	1.23 (0.83-1.83)	0.313	1.52 (0.85-2.72)	0.159
Four	1.70 (1.16-2.49)	0.006	2.39 (1.17-4.90)	0.017
Five	1.55 (1.05-2.27)	0.027	2.28 (0.98-5.31)	0.057
**Area and socioeconomic status (SES)**				
Urban, high SES	17.55 (9.98-30.88)	< 0.001	18.10 (10.24-32.00)	< 0.001
Urban, middle SES	8.94 (5.03-15.90)	< 0.001	8.52 (4.65-15.60)	< 0.001
Urban, low SES	2.70 (1.44-5.08)	0.002	2.55 (1.29-5.02)	0.007
Rural, low/disadvantaged SES	Reference	-	Reference	-

^a^The model is adjusted for age and gender

**Figure 4:**
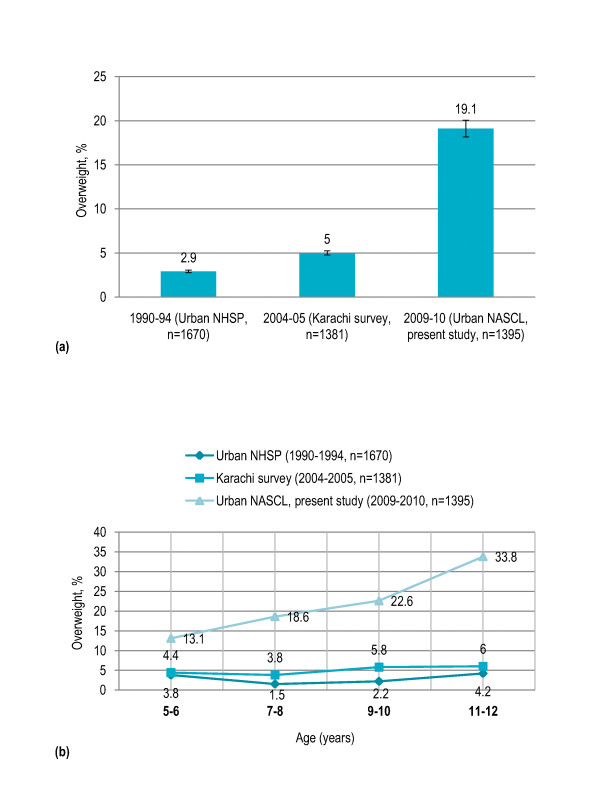
**Trend in prevalence of overweight among urban primary school children in Pakistan; (a) shows over-all trend, and (b) shows age-specific trend**. Note: US CDC 2000 child growth reference (defining overweight as >85^th ^percentile BMI-for-age) was used in the previous studies, and results from urban sample in the present study are presented with respect to the same reference for purpose of trend analysis.

In Pakistan, previous studies have reported the prevalence of overweight among urban primary school children aged 5-12 years as 3% (urban NHSP, 1990-94, n = 1670) and 5% (Karachi survey, 2004-05, n = 1381) that increased to 19% in the urban sample of present study (NASCL, 2009-10, n = 1395) [Figure 4 (a)]. An increasing trend in prevalence of overweight with age was observed in previous studies and it was significantly more pronounced in the present study [Figure 4 (b)].

## Discussion

Prevalence of overweight and obesity among Pakistani primary school children aged 5-12 years was 17% and 7.5% respectively. There was four-fold increase in overweight school-aged children in the past five years in the urban Pakistan that highlights the alarmingly rapid rise in childhood overweight and obesity. This pattern is consistent with global trends in childhood obesity observed in the developed countries like United States [29,30]. A potential public health issue for the developing countries is the rapidly increasing childhood obesity leading to emerging epidemic of NCDs, which in turn will create an enormous socioeconomic and public health burden in coming decades [9-11,31,32]. Worldwide, NCDs account for about 60% deaths annually and the disease burden is currently greatest and continuing to grow in the developing countries where 66% of these deaths occur [2].

Prevalence of overweight by the IOTF cut-offs was twice the prevalence by the WHO 2007 reference (33% versus 17%) and prevalence of obesity by the IOTF cut-offs was three times higher than that calculated by the WHO 2007 reference (24% versus 7.5%). Using IOTF cut-offs for overweight and obesity in Pakistani school-aged children would result in higher estimates than the WHO 2007 reference. In Pakistan, present study is the first to report prevalence statistics by the WHO 2007 and IOTF cut-offs.

More boys were overweight than girls but the association was not statistically significant, however, obesity prevalence was significantly higher among boys than girls in line with the results reported in urban India, Brazil, Finland, Canada, and Asian-Americans in United States [33-37]. Grade- and gender- specific prevalence trend showed that more boys were overweight in grades one and two while more girls were overweight in grades three to five. Moreover, the mean BMI was higher in boys in grades one and two as compared to girls while in grades four and five a higher BMI in girls was observed. Higher prevalence among girls in higher grades may be due to genetic factors and pubertal growth spurt. More boys were overweight in the urban area with high SES where highest obesity prevalence was observed. A possible explanation for higher body fatness among boys might be the socio-cultural matrix in South Asia where parents prioritize boys, especially in the younger age groups, in feeding practices. Parents are less likely to encourage sons to lose weight, perhaps because of the larger and more muscular ideal male body shape [38]. Prevalence of overweight showed a significantly increasing trend with grade and age and higher grade independently predicted the risk of being overweight. The findings are consistent with previous studies in Pakistan and elsewhere [21,23,39,40].

Living in the urban area with higher SES was strong independent predictor of being at risk for overweight. Higher obesity prevalence was reported in the Pakistani adults living in urban areas and having high SES [40]. Increased prevalence of overweight with urbanization have been reported both in the developing and developed countries [21,29,41,42]. Higher prevalence of overweight with high SES was observed in the present study, in contradiction to higher prevalence of overweight with low SES observed previously in the developed countries like United States and developing countries like Brazil [34,41,43]. Different socio-cultural circumstances in South Asia explain the contradiction and studies in India have indicated the same trend with prevalence increasing with higher SES [44,45]. In the developing countries, gross national product (GNP) has been associated positively with overweight among pre-school children [46]. Prevalence of overweight was higher in private schools than public schools, consistent with a study in Brazil [47].

Urbanized lifestyle including reduced physical activity and increased sedentary living and unhealthy diets high in saturated fats, sugar and refined foods are the probable causes of the emerging childhood obesity epidemic in the developing countries undergoing nutrition transition [29,32,48]. Increasingly obesogenic environments reinforced by many of the cultural changes associated with globalization further aggravate the situation, especially among children and adolescents [32]. Efforts to stop childhood obesity should be made on all fronts and targeted interventions, tailored to local circumstances and involving communities, should begin early in life [13,14,20].

Prevention and treatment efforts, following a global approach with proper monitoring and implementation, are effective and there is little evidence of negative effects, either physiological or psychological [22,49,50]. Suggested approaches include labeled diets, exercise sessions and increased general lifestyle activity combined with the use of behavioral change methods [50]. Family-based interventions are routinely recommended for obese school-aged children and school-based programs are recommended for those at risk of being overweight and obese [49]. Rapidly increasing rates of obesity among school-aged children in Pakistan suggest the urgent need for a primary prevention program [51]. A National preventive strategy for childhood obesity should be developed and a pilot preventive program should be initiated. Currently, School Health and Nutrition Supervisors working in Pakistan's National Maternal, Newborn, and Child Health (MNCH) Program can be used for growth monitoring and BMI tracking in school-going children.

Cross-sectional nature of the study should be considered when interpreting the findings. Future longitudinal studies involving these factors are therefore warranted to establish the temporal nature and causality of these associations. Although data collection followed a standard protocol, digital scales were not used. Variability in the data ascertainment may have introduced error into the prevalence estimates; however, we do not anticipate large or systematic differences. The findings can be generalized to South Asian primary school children, who share the same genetic and environmental factors with the sample. We speculate that overweight may be a greater issue in a greater number of countries among school-aged children and countries contemplating nutrition surveys ought to consider including school-aged children.

## Conclusion

Alarmingly rapid rise in overweight and obesity among Pakistani primary school children was observed, especially among the affluent urban population. The findings support the urgent need for National preventive strategy for childhood obesity and targeted interventions tailored to local circumstances with meaningful involvement of communities.

## Competing interests

The authors declare that they have no competing interests.

## Authors' contributions

All authors contributed significantly in all phases of the study in accordance with uniform requirements established by the International Committee of Medical Journal Editors. All authors read and approved the final manuscript.

## Pre-publication history

The pre-publication history for this paper can be accessed here:

http://www.biomedcentral.com/1471-2458/11/724/prepub
