# Correction: prevalence and socioeconomic correlates of overweight and obesity among Pakistani primary school children

**DOI:** 10.1186/1471-2458-12-532

**Published:** 2012-07-20

**Authors:** Muhammad Umair Mushtaq, Sibgha Gull, Hussain Muhammad Abdullah, Ubeera Shahid, Mushtaq Ahmad Shad, Javed Akram

**Affiliations:** 1 Ubeera Memorial Research Society, Allama Iqbal Medical College, Lahore, Punjab 54000, Pakistan; 2 District Health Office Nankana Sahib, Punjab Department of Health, Nankana Sahib, Punjab 39100, Pakistan; 3 King Edward Medical University, Lahore, Punjab, 54000, Pakistan

## Text

Since the publication of our article [1], we have noticed some errors in the final published version, for which the corresponding author accepts full responsibility. Page references are to the final PDF version.

### Page 3: Results, second paragraph

Lines 1-2: "BMI… 20.7 (5.02) kg/m^2^…" *should read* "BMI… 16.0 (3.0) kg/m^2^…”

Lines 6-8: “According to the IOTF cut-offs, overweight and obesity prevalence was 33 % (95 % CI 31.1-35.3) and 24 % (95 % CI 22.4-26.2) respectively” *should read* “According to the IOTF cut-offs, overweight and obesity prevalence was 8.3 % (95 % CI 7.1-9.6) and 4.7 % (95 % CI 3.8-5.7) respectively”

### Page 4: Table 1

**Table 1:** Mean and standard deviation (SD) for height, weight and BMI of primary school children in Lahore, Pakistan (n = 1860)

**Characteristics**	**n**	**Height (cm)**	**Weight (kg)**	**BMI (kg/m**^**2**^**)**
**Boys (n = 977)**				
5 years (61-71 months)	84	113.7 (7.3)	19.9 (4.6)	15.2 (2.1)
6 years (72-83 months)	161	118.3 (5.9)	21.6 (5.0)	15.3 (2.8)
7 years (84-95 months)	160	122.9 (8.0)	23.5 (5.1)	15.5 (2.4)
8 years (96-107 months)	158	128.7 (7.6)	26.9 (5.9)	16.1 (2.5)
9 years (108-119 months)	161	134.2 (8.1)	29.7 (7.6)	16.4 (3.1)
10 years (120-131 months)	147	138.4 (8.0)	33.3 (9.5)	17.2 (3.5)
11 years (132-143 months)	69	138.6 (7.7)	31.8 (6.8)	16.5 (2.7)
12 years (144-155 months)	37	140.0 (8.3)	31.8 (7.3)	16.1 (2.3)
**Girls (n = 883)**				
5 years (61-71 months)	72	115.4 (7.3)	19.3 (3.2)	14.4 (1.5)
6 years (72-83 months)	143	119.1 (7.6)	21.0 (4.9)	14.7 (2.4)
7 years (84-95 months)	157	124.0 (6.3)	24.0 (5.5)	15.5 (2.7)
8 years (96-107 months)	159	128.1 (7.1)	26.4 (6.8)	15.9 (2.9)
9 years (108-119 months)	151	133.3 (7.8)	30.4 (8.2)	17.0 (3.5)
10 years (120-131 months)	120	138.4 (9.3)	33.3 (10.1)	17.2 (3.8)
11 years (132-143 months)	62	143.3 (9.6)	36.5 (11.0)	17.5 (3.7)
12 years (144-155 months)	19	146.0 (9.4)	36.4 (9.9)	16.9 (3.3)

The values for mean and standard deviation (SD) for BMI (kg/m^2^) are revised.

### Page 4: Table 2

The values for mean BMI (SD) and overweight and obesity prevalence according to the IOTF cut-offs are revised.

**Table 2:** Prevalence of overweight and obesity among primary school children in Lahore, Pakistan (n = 1860)

			**WHO 2007**		**IOTF**	**IOTF**
**Characteristics**	**n**	**Mean BMI (SD)**	**% (95 % CI)**	**Mean BMI-for-age z-score (SD)**	**% (95 % CI)**	
Severely obese	36	25.7 (2.9)	1.9 (1.3-2.6) ^a^	3.7 (0.7)		
Obese	140	23.4 (2.8)	7.5 (6.3-8.7) ^b^	2.8 (0.7)	4.7 (3.8-5.7) ^d^	
Overweight	316	21.2 (2.9)	17.0 (15.4-18.8) ^c^	2.0 (0.8)	8.3 (7.1-9.6) ^e^	
Total sample	1860	16.0 (3.0)		-0.3 (1.5)		

^a^ > +3SD, ^b^ > +2SD, ^c^ > +1SD of World Health Organization (WHO) 2007 BMI-for-age reference.

^d, e^International Obesity Task Force (IOTF) cut-offs for obesity and overweight respectively.

### Page 5: Figure 1

**Figure 1:**
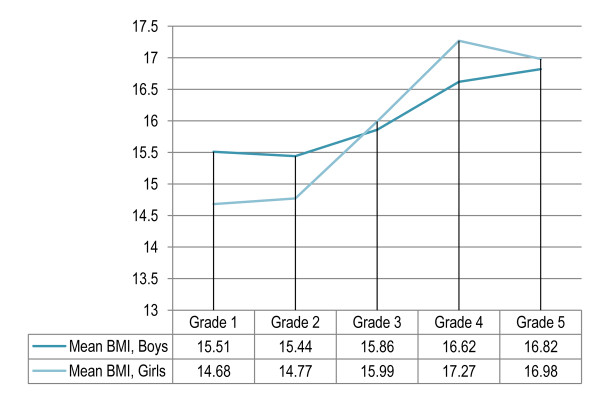
Grade- and gender- specific mean BMI among primary school children in Lahore, Pakistan (n = 1860).

The values for grade- and gender- specific mean BMI are revised.

### Page 6: Second paragraph

Lines 1-8: “Prevalence of overweight by the IOTF cut-offs was twice the prevalence by the WHO 2007 reference (33 % versus 17 %) and prevalence of obesity by the IOTF cutoffs was three times higher than that calculated by the WHO 2007 reference (24 % versus 7.5 %). Using IOTF cut-offs for overweight and obesity in Pakistani schoolaged children would result in higher estimates than the WHO 2007 reference.” *should read* “Prevalence of overweight by the IOTF cut-offs was half the prevalence by the WHO 2007 reference (8 % versus 17 %) and prevalence of obesity by the IOTF cutoffs was two-third of that calculated by the WHO 2007 reference (5 % versus 7.5 %). Using IOTF cut-offs for overweight and obesity in Pakistani schoolaged children would result in lower estimates than the WHO 2007 reference. A relatively lower overweight and obesity prevalence with use of the IOTF cut-offs as compared to the WHO reference had been reported elsewhere [2,3].”

In present study, the estimates for overweight included obese children.

## Competing interests

The authors declare that they have no competing interests.

## Authors' contributions

All authors contributed significantly in all phases of the study in accordance with uniform requirements established by the International Committee of Medical Journal Editors. All authors read and approved the final manuscript.

## Pre-publication history

The pre-publication history for this paper can be accessed here:

http://www.biomedcentral.com/1471-2458/12/532/prepub
